# Relations Between L2 Proficiency and L1 Lexical Property Evaluations

**DOI:** 10.3389/fpsyg.2022.820702

**Published:** 2022-03-17

**Authors:** Elif Altın, Nurdem Okur, Esra Yalçın, Asude Firdevs Eraçıkbaş, Aslı Aktan-Erciyes

**Affiliations:** ^1^Department of Psychology, Kadir Has University, İstanbul, Turkey; ^2^Department of Psychology, 29 Mayıs University, İstanbul, Turkey; ^3^Department of Translation and Interpreting Studies, Boğaziçi University, İstanbul, Turkey

**Keywords:** bilingualism, imageability, concreteness, frequency, L2 proficiency

## Abstract

The present study investigates the relations between L2-English proficiency and L1-Turkish lexical property evaluations. We asked whether L2 proficiency affects lexical properties, including imageability and concreteness ratings of 600 Turkish words selected from the Word Frequency Dictionary of Written Turkish. Seventy-two participants (L1-Turkish - L2-English) provided ratings of concreteness and imageability for 600 words on a 7-point scale. In order to assess their L2 proficiency, we administered Peabody Picture Vocabulary Test-IV (PPVT-IV). We divided categories into two subcategories as high and low for the frequency, concreteness, imageability, and age of acquisition (AoA). The relationship between these subcategories and imageability-concreteness was examined by mixed effects linear regression analyses. We found that L2 proficiency and imageability ratings were positively correlated and specifically, this positive association was evident for low-frequency words and later acquired words. Results are in line with the interaction of bilingual representation under the dual-coding theory which suggests that bilinguals develop an interconnected imaginal representation for two languages as opposed to separate verbal representations. As L2 proficiency increased, the imageability also increased. These findings have implications for literature investigating the relationship between L2 proficiency and linguistic outcomes. Additionally, findings point to the importance of considering the L2 proficiency of participants when lexical tasks that involve cue words or word lists are used.

## Introduction

Cognitive tasks that require a certain level of lexical processing have always played a critical role in studies of psychology and psycholinguistics. To name a few, tasks such as lexical decision, semantic organization, picture naming, word recognition along with word evaluation tasks such as word rating have been widely used in the field (Meyer and Schvaneveldt, 1971; Forster and Chambers, 1973; Johnson et al., 1996). However, when working with verbal stimuli, there may be a variety of factors that can result in processing differences and in turn influence the overall performance. Hence, that is why psycholinguistic variables such as frequency (Forster and Chambers, 1973), word length (Baddeley et al., 1975), concreteness (Walker and Hulme, 1999), and imageability (Paivio, 1971) have drawn a special interest of researchers throughout the past decades. There is a considerable amount of information regarding how these lexical properties may affect task performance and in which ways they may be related to one another. However, no study has taken into account how second language (L2) proficiency might affect evaluations of these lexical properties.

Previous research suggest that words with higher frequency are processed faster or more accurately on a wide variety of different tasks compared to low-frequency words due to the fact that lexical access for those items is easier (Carroll and White, 1973; Forster and Chambers, 1973; Whaley, 1978). Same is true for words that are acquired at an earlier age compared to later acquired ones (Gilhooly and Logie, 1980; Morrison and Ellis, 1995; Bonin et al., 2002) and for more concrete words (Paivio, 1971; James, 1975; de Groot et al., 1994) as well as words that are more imageable (Holmes and Langford, 1976; Schwanenflugel et al., 1988; Cortese and Schock, 2013). In other words, those words, respectively, hold an advantage to be accessed and processed easier than less frequently used, later learned, abstract, and less imageable ones. Furthermore, several lines of evidence demonstrate that these lexical properties, including concreteness, frequency, and imageability are so closely related that it is often challenging to measure and distinguish the effect of one variable from the other (Gernsbacher, 1984; Göz et al., 2017). For instance, Morrison and Ellis (1995) argue that earlier studies that showed frequency effects (i.e., faster processing of commonly used words) demonstrated such results as they failed to control for AoA. The two variables (i.e., frequency and AoA) are highly correlated since words acquired earlier are also commonly used later in life (Gerhand and Barry, 1998). Similarly, concreteness and imageability are also two variables that have been almost used interchangeably by many researchers due to their high correlation as concrete items are easier to evoke an image in mind than abstract items (McMullen and Bryden, 1987; Fliessbach et al., 2006; Reilly and Kean, 2007). Besides, AoA can also pose as a confounding variable for concreteness and imageability measures based on the fact that early acquired words are mostly concrete, and hence, mostly imageable (Bird et al., 2001; Bonin et al., 2004; Göz et al., 2017).

According to the aforementioned literature, it has been illustrated that lexical properties can critically influence speed and accuracy, and additionally can affect one another during word processing. In light of this information, it can be concluded that these variables need to be controlled for to ensure the internal reliability of the experiment and have a better comprehension of the results. Traditionally, it has been an established practice to measure and control these variables. For that reason, a considerable amount of normative studies has collected norms for frequency (Desrochers and Thompson, 2009), AoA (Davies et al., 2016), concreteness, and imageability (Yao et al., 2018) ratings of a large set of verbal material across different languages. However, while paying close attention to the potential effect of these properties, one important factor that often goes overlooked is the prevalence of bilingualism. In a world where almost half of the population is bilingual (Grosjean and Li, 2013), still very little is known about whether or to what degree these lexical properties in the first language (L1) can be influenced by L2 knowledge. From bilingualism studies that examine bidirectional transfer between languages, we know that L2 can have an impact on L1 lexicon and semantics (van Hell and Dijkstra, 2002; Pavlenko, 2003) as well as conceptual representations (Shimron and Chernitsky, 1995; Kecskes and Papp, 2003) even in late bilinguals. In understanding bilingual lexicon, the Distributed Feature Model (De Groot et al., 1994) suggests that bilinguals translate concrete words faster than abstract ones (Kroll and Stewart, 1994; Van Hell and De Groot, 1998). This implies that representations of concrete words are shared largely across languages, while representations of abstract words share fewer semantic elements. As the L2 proficiency increases, the links between L2 words and concepts become stronger (Potter et al., 1984). A more recent model, the Shared Asymmetrical Model (Dong, et al., 2005) suggests that there are two separate stores of L1- and L2-specific conceptual items, as well as a common large conceptual store shared by these stores of L1 and L2. This model suggests that some concepts might be more salient in some languages compared to others. For instance, they propose that the concept of “color” *red* might be common for both English and Chinese, whereas the concept of “danger” would be more salient in English word *red* than in than in the Chinese word *hóngsè,* and concept of *“bride”* will be more salient in Chinese word *hóngsè* than in English word *red*. In their empirical study, Dong, et al. (2005) suggested that L1-Chinese L2-English bilinguals’ conceptual representations for Chinese words were different compared to monolingual Chinese individuals. Thus, adding an L2 to L1 had projections on L1 conceptual-lexical organization. Finally, the Modified Hierarchical Model (Pavlenko, 2009) adds the developmental dimension (i.e., developmental sequence from lexical to conceptual mediation in acquiring L2) to the picture which is closely related with L2 proficiency. Consequently, L2 proficiency may be another variable to consider along with lexical properties in word processing tasks. In the present study, we ask how these lexical properties (i.e., frequency, concreteness and imageability, AoA) are related to one another and how L2 proficiency affects this relationship. Our research aims to fill the gap in the literature by investigating how and to what degree L2 proficiency may have an influence on concreteness and imageability ratings of words in L1 taking frequency and AoA into account.

### Relationship Between L2 Proficiency, Concreteness and Imageability

Concreteness and imageability are the two main terms that refer to the image evoking property of lexical material. Generally, in word evaluation tasks, concreteness is defined as “the extent to which the item can be experienced by senses” while imageability is defined as “the extent to which the item evokes a mental image” (Richardson, 1975, p. 235). For instance, concreteness effect, which can be defined as the faster and more accurate processing of concrete words, can be observed in a large variety of different tasks including memory tasks (Walker and Hulme, 1999; Cortese et al., 2015), and lexical decision tasks (Schwanenflugel et al., 1988; Cortese and Schock, 2013). Although, some studies used the concreteness and imageability terms interchangeably due to their similarities (Fliessbach et al., 2006; Reilly and Kean, 2007) the two concepts are in fact dissociable despite the positive correlation between them (Paivio et al., 1968). As Richardson (1975) argues that they cannot be used alternatively to reflect the image-evoking property of words, as concreteness may rather be “just a feature of lexical organization,” many recent studies are also in the same line, arguing that the two concepts are distinct as concreteness reflects word’s degree of perceptibility while imageability refers to image arousing capacity (Connell and Lynott, 2012; Brysbaert et al., 2014).

One of the theoretical approaches that attempts to explain the concreteness effect which might also be related to L2 proficiency is the Dual-coding theory (Paivio, 1971). The Dual coding theory suggests a representational system in which there are two separate but related systems: while one processes verbal material, the other processes imaginal material. Consequently, concrete words which are believed to have both verbal and imaginal properties have representations in both verbal and imagery systems in contrast with abstract words which only have verbal representations. As a result, the concreteness effect occurs due to the fact that one-to-many mapping is more powerful than one-to-one mapping during lexical processing (Altarriba et al., 1999). In other words, additional mapping for concrete items eases the access. Furthermore, according to the Dual-coding theory, bilingual individuals have acquired different verbal systems for different languages while only having one imagery system connected to them. This implies that a bilingual’s imagery system might be more enhanced as there is more than one verbal representation which an imaginal representation can connect with, potentially leading to a higher imageability of words especially in their native language in which the link between words and their representations is stronger.

Although this theory has been widely known in the field for over five decades, to the best of our knowledge, there are only a few studies that examine whether concreteness effect is more pronounced in bilinguals than monolinguals (Paivio and Desrochers, 1980; Ransdell and Fischler, 1989). While Paivio and Desrochers (1980) suggest a concreteness advantage for bilinguals, (Ransdell and Fischler, 1989) in their experiments find no evidence to support this claim. Surprisingly, despite the importance of the abovementioned implication of the theory, no previous study has investigated how L2 proficiency may influence the perceived imageability as well as concreteness of words in L1.

### Relationship Between L2 Proficiency and Age of Acquisition and Frequency

Word frequency, among other factors that are associated with word processing efficiency, has drawn the most attention in the literature. It is now well established by a number of studies that word frequency effect is reflected in lexical decision tasks (Balota and Chumbley, 1984), word naming tasks as well as a number of memory tasks (Brysbaert et al., 2018) in many different languages (e.g., Desrochers and Bergeron, 2000; Alonso et al., 2011). Similarly, AoA can also be a reliable predictor of the processing speed and accuracy in word naming (Gilhooly and Logie, 1981; Gerhand and Barry, 1998; Elsherif et al., 2019), lexical decision tasks (Morrison and Ellis, 1995; Arnon et al., 2017) and memory tasks (Morrison and Conway, 2010). However, frequently used words are also typically acquired earlier in life which leads to a strong correlation between the two variables. For that reason, some researchers put particular emphasis on the possibility that the frequency effect demonstrated in these tasks can merely be an outcome of AoA effect (Morrison and Ellis, 1995; Gerhand and Barry, 1998; Izura et al., 2011). Nevertheless, there are a considerable amount of studies that prove otherwise by demonstrating the two effects independently when the correlated properties are controlled (Butler and Hains, 1979; Barry et al., 1997; Brysbaert et al., 2016). Additionally, data from several studies suggest an interaction between the two which implies that AoA can be related with low-frequency words but not with high-frequency words (Bonin et al., 2002; Brysbaert et al., 2016). In sum, although being related, AoA and frequency have independent influences in lexical processing.

There is a growing body of literature that recognizes the importance of examining frequency effect among bilinguals (Duyck et al., 2008;Gollan et al., 2008; Ivanova and Costa, 2008). In one of the studies that focused on frequency effect in word production in a bilingual context, Gollan et al. (2008) used picture naming tasks to examine the frequency effect demonstrated by English-Spanish bilinguals and English monolinguals. Data from the study have identified a larger frequency effect in bilinguals as opposed to monolinguals, especially for low-frequency words. The authors explained these results in light of the weaker links hypothesis (Gollan et al., 2008). According to weaker links hypothesis, bilinguals divide their language use between two languages which consequently leads to using words in each language less often than monolinguals do. This decreased frequency-of-use causes weaker links between semantics and phonology, as the less you produce and comprehend a word the harder it is to access. Furthermore, low-frequency words are more likely to be influenced by the differences of usage between bilinguals and monolinguals than high-frequency words since the links are even weaker for low-frequency words. In the same vein, Duyck et al. (2008) demonstrated a larger frequency effect in bilinguals’ L2 as opposed to their L1. Yet, when compared, in a second experiment, bilinguals showed a frequency effect in their L1-Dutch similar to the effect displayed by monolinguals in English. In contrast with these findings, other researchers reported that bilinguals demonstrated a larger frequency effect in their native language than monolinguals did. (Gollan et al., 2008; Ivanova and Costa, 2008). In sum, literature shows that bilinguals and monolinguals differ in terms of frequency effect which might be explained by the weaker links hypothesis.

One of the implications of Gollan’s findings and weaker links hypothesis for the present study is that any kind of change to any lexical property among bilinguals will be more pronounced for the low-frequency words since links are weaker and thus more adept to change than high-frequency words which may have stronger links. In terms of AoA, the same might be expected for later acquired AoA words, as they might have weaker links compared to earlier acquired AoA words. Thus, along the same lines, if a change in perceived lexical properties such as imageability can be seen in bilinguals’ L1, it is more likely to be manifested in later acquired words rather than early acquired ones.

### The Present Study

In light of the literature reviewed here, we asked how lexical properties represented in frequency and AoA are related to subjective ratings of concreteness and imageability. We are specifically interested in how L2 proficiency affects this relationship. Previous studies often neglect the role of L2 on such evaluations in a world where bilingualism is widespread. This study aims to examine whether L2 learning provides a more enriched world view for bilinguals. We hypothesized that L2 proficiency would lead to more enriched processing in line with the Dual-coding theory reflected higher imageability evaluations, depending on close relations between imageability and concreteness, our first prediction is that imageability and concreteness ratings will be positively associated with the L2 proficiency in line with the Dual-coding theory. As imageability and concreteness are often found to be related, we will also investigate the unique relation between L2 proficiency and imageability controlling for concreteness as well as the unique relation between concreteness and L2 proficiency controlling for imageability. Secondly, we expect that the relation between imageability ratings and L2 proficiency will be more pronounced compared to the association between concreteness and L2 proficiency. The reason for this expectation is our assumption that imageability ratings might be more subjective than concreteness ratings. Both concepts are closely related; however, on the one hand concreteness is more a feature of lexical organization, on the other hand imageability reflects the imaginal processes, and therefore more open to change by L2 proficiency (Richardson, 1975). For low-frequency words, we expected the relation between imageability and L2 proficiency to be higher compared to high-frequency words. Lastly, we predict that the relation between imageability and L2 proficiency will be more pronounced for later acquired words (AoA > 6) compared to earlier acquired words (AoA < 4). We expect to see these differences since low-frequency words are usually required later in life, and both low-frequency words and later acquired words are more open to the influence of second language.

## Materials and Methods

### Participants

Seventy-two undergraduate students (83% female) between 19 and 27 years old (*M* = 20.81, SD = 1.74) participated in the study for course credit. All our participants knew English as their L2 and were enrolled in English-taught courses. They practiced English as their main language in their education. The Ethics Committee of [Blinded] University approved the present study (Ethical approval number: 17446481-050.06.04-E.2034).

### Materials

We used 600 Turkish words from Turkish Word Norms (Tekcan and Göz, 2005) which provides the imageability and concreteness ratings of the words upon completion of a norming study by 100 university students on a 1-to-7 point scale. The word frequency figures of 600 words were provided from the Word Frequency Dictionary of Written Turkish (Göz, 2003). The 600 words consist of both concrete (51%) and abstract (49%) words which are mostly nouns (88%), and the rest are adjectives (12%). The words were selected as 200 words of all types in the form of low (≤20 per million), medium (50–99 per million), and high-frequency (≥100 per million) words (Tekcan and Göz, 2005).

#### Lexical Property Evaluation Task

Lexical property evaluation task was administered online with survey prepared *via* Qualtrics^®^. Words were given in completely counterbalanced order, in two blocks, each containing 300 words. The purpose of having two separate blocks was to avoid mental fatigue and allow participants rest in between the two blocks. Participants first evaluated the concreteness and then imageability of 300 words on a 1-to-7 point scale, and in the second block, they completed the ratings for the rest of the 300 words. At the beginning of the second block, instructions were given again.

#### Language Task

In order to assess participants’ L2 English proficiency, Peabody Picture Vocabulary Test (PPVT)–IV was administered. The test intends to measure receptive vocabulary skills in English which is an indicator of proficiency. The test consists of 228 test items. Four pictures were shown on each page in the test, and participants were asked to tell the number of the correct picture according to the word they heard. The test was adapted into an online format, where all words were recorded by a single research assistant, thus participants listened the same voice for different words. All stimuli were shown on a screen *via* the Zoom Platform. Scoring was completed online by the research assistant.

#### Demographic Form

A demographic-linguistic form was provided to the participants in order to gather more information about L2 practices, including L2 acquisition age, and the number of years studying L2. The form was completed online by participants upon completion of Lexical Property Evaluation Task and PPVT-IV.

Descriptive information for participants with respect to their L2 exposure, L2 age of onset (AoO), i.e., the information regarding when they first exposed to L2-English and Peabody Picture Vocabulary Test- IV (PPVT-IV) scores are represented in Table 1.

**Table 1:** Means and standard deviations of L2 exposure, age of onset (AoO), and PPVT-IV scores.

Mean AoO	% of participant	L2 Exposure (years) Mean (SD)	PPVT-IV	Mean(SD)	Min	Max
5–9	31.94	13.17(2.17)	117.70(33.97)	67	163
10–14	54.79	9.85(2.73)	103.64(26.72)	43	154
15–18	12.5	4.33(2.23)	83.00(20.40)	49	121

### Procedure

Participants were first provided the link for Lexical Property Evaluation Task. All participants completed concreteness and imageability ratings for 600 words which totaled up to 1,200 evaluations. After completing 300 words, participants were allowed to take a 15-min break and continued the rest of the 300 words. Completion of the survey took around 1.5 h.

Upon completion of the survey, participants were invited to an online session where PPVT-IV was administered. Research assistants administered PPVT-IV involved by displaying test items on screen while giving instructions. For each item displayed, research assistants took notes of the answers by the participants in record forms. Each session of PPVT-IV administration took approximately 20–30 min. Upon completion of PPVT-IV administration, participants were sent a link where they were asked to complete the online demographic form prepared *via* Qualtrics^®^.

## Results

In order to examine the relation between L2 proficiency and L1 lexical property evaluations, as preliminary analyses, we first computed zero-order correlations. Our main analyses involved linear mixed effects model investigating the relation between L2 proficiency and lexical properties. We ran two models using linear mixed-effects in R (R Core Team, 2021), taking lexical property ratings (for imageability and concreteness as dependent variables). One model investigated relations for L2 Proficiency, Frequency, Concreteness, and Imageability, and the other model for L2 Proficiency, AoA, Concreteness, and Imageability. We did not introduce AoA and frequency in the same model simultaneously for two reasons: First, AoA and frequency are two variables that are closely related, which might have caused multicollinearity. Second, introducing high/low AOA and high/low-frequency information into the model simultaneously would cause a smaller number of words left, thus only intersecting words would remain (for instance, neither and nor high/low AoA words would be omitted although they might be high or low frequent). The details of the models tested are given in section “Relations Between L2 Proficiency, AoA, Frequency, and Lexical Evaluations”.

### Data Preparation

For correlations, we computed a total of 16 different subscores (high/low) for subjective ratings of concreteness and imageability (8 subscores for each) based on previous measures of frequency, concreteness, imageability derived from Turkish word norms (Tekcan and Göz, 2005) and AoA from Göz et al. (2017). For instance, we computed 8 subscores of subjective ratings of concreteness based on high/low frequency, high/low concreteness, high/low imageability, and high/low AoA. We computed the same subscores for imageability ratings as well.

#### High/Low-Frequent Words

Following Tekcan and Göz (2005), words are categorized as low-frequency words if the frequency for the word is less than 20 and as high-frequency words, if the frequency of the word is over 100. Within the 600 words, there are 153 words (25.5%) in the low-frequency group and 201 words (33.5%) in the high-frequency group.

#### High/Low Imageable Words

In the 7-point scale, the midpoint “4” corresponded to neither imageable / nor nonimageable, and so we categorized those less than or equal to “3” as low imageable, and those greater than or equal to “5” as high imageable (Tekcan and Göz, 2005). There are 90 words (15%) in the low imageability group and 265 words (44.2%) in the high imageability group.

#### High/Low Concrete Words

Similar to imageability, depending on concreteness, words are divided into two groups (“4” correspond to neither concrete/nonconcrete, less than or equal to “3” to low concrete, and greater than or equal to “5” to high concrete; Tekcan and Göz, 2005). There are 147 words (24.5%) in the low concreteness group and 337 words (56.2%) in the high concreteness group.

#### High/Low AoA Words

In two groups of age-of-acquisition (AoA); there are 170 words (28.3%) in the low AoA group (age of <4) and 48 words (8%) in the high AoA group (age of >7).

### Preliminary Analyses

We performed a paired samples t-test in order to compare imageability ratings of high vs. low imageable words. There was a significant difference in the ratings for high imageability words (*M* = 6.45, SD = 0.55) and low imageability words (*M* = 2.62, SD = 1.02); *t*(71) = 27.45, *p* < 0.001. Additionally, we ran paired samples t-test to compare concreteness ratings of high vs. low concrete words. There was a significant difference in the scores for high concrete words (*M* = 6.11, SD = 0.60) and low concrete words (*M* = 2.28, SD = 0.94); *t*(71) = 25.95, *p* < 0.001. Thus we confirmed that high imageable words were rated more imageable than low imageable words. Additionally, high concrete words were rated as more concrete than low concrete words. Descriptive statistics of PPVT-IV and all main and subscores of imageability and concreteness ratings are represented in Table 2.

**Table 2:** Descriptive values of PPVT-IV and all main and subscores of imageability and concreteness ratings.

	Mean (SD)	Min	Max
PPVT	105.71(30.54)	43	199
Overall imageability	4.83(0.64)	3.19	6.23
hi_freq_image	5.16(0.63)	3.49	6.52
lo_freq_image	4.40(0.68)	2.71	5.87
hi_conc_image	6.12(0.56)	3.84	6.82
lo_conc_image	2.64(1.12)	1.00	5.37
hi_image_image	6.46(0.55)	4.07	7.00
lo_image_image	2.63(1.02)	1.09	5.21
hi_AoA_image	3.78(0.79)	1.96	5.48
lo_AoA_image	5.99(0.53)	3.95	6.83
Overall concreteness	4.72(0.52)	3.52	5.64
hi_freq_concrete	5.01(0.52)	3.81	6.06
lo_freq_concrete	4.38(0.63)	2.97	5.71
hi_conc_concrete	6.11(0.60)	3.91	6.90
lo_conc_concrete	2.28(0.94)	1.02	5.01
hi_image_concrete	6.37(0.60)	3.89	6.95
lo_image_concrete	2.50(0.90)	1.17	5.04
hi_AoA_concrete	3.86(0.76)	2.10	5.38
lo_AoA_concrete	5.85(0.52)	4.05	6.56

### Relations Between L2 Proficiency, AoA, Frequency, and Lexical Evaluations

We asked whether L2 proficiency reflected in PPVT-IV is associated with L1 lexical property evaluations for imageability and concreteness ratings. In order to investigate this question, as a preliminary analysis, we first computed zero-order Pearson correlations between L2 proficiency, concreteness, and imageability ratings. The results indicated that overall concreteness ratings were not correlated with L2 proficiency, *r*(71) = 0.11, *p* = 0.39. On the other hand, imageability ratings were correlated with L2 proficiency, *r*(71) = 0.27, *p* = 0.03. There was also a correlation between imageability and concreteness, *r*(71) = 0.63, *p* < 0.001. We then computed zero-order correlations between L2 proficiency and all subcategories of lexical property ratings (see Tables 3, 4 for details).

**Table 3:** Correlation between PPVT-IV and main and subscores of concreteness ratings.

	1	2	3	4	5	6	7	8	9	10
1. PPVT-IV	1									
2. Overall concreteness	0.102	1								
3. hi_freq_concrete	0.091	0.966^**^	1							
4. lo_freq_concrete	0.089	0.943^**^	0.836^**^	1						
5. hi_conc_concrete	0.068	0.624^**^	0.682^**^	0.524^**^	1					
6. lo_conc_concrete	0.076	0.537^**^	0.427^**^	0.578^**^	−0.295^*^	1				
7. hi_image_concrete	0.039	0.466^**^	0.564^**^	0.330^**^	0.967^**^	−0.444^**^	1			
8. lo_image_concrete	0.091	0.589^**^	0.457^**^	0.664^**^	−0.235^*^	0.975^**^	−0.411^**^	1		
9. hi_AoA_concrete	0.118	0.849^**^	0.726^**^	0.930^**^	0.291^*^	0.680^**^	0.088	0.779^**^	1	
10. lo_AoA_concrete	0.047	0.708^**^	0.788^**^	0.566^**^	0.964^**^	−0.159	0.935^**^	−0.128	0.333^**^	1

* *Correlation is significant at the 0.05 level (2-tailed)*.

** *Correlation is significant at the 0.01 level (2-tailed)*.

**Table 4:** Correlation between PPVT-IV and main and subscores of imageability ratings.

	1	2	3	4	5	6	7	8	9	10
1. PPVT-IV	1									
2. overall imageability	0.265^*^	1								
3. hi_freq_image	0.217	0.979^**^	1							
4. lo_freq_image	0.300^*^	0.938^**^	0.863^**^	1						
5. hi_conc_image	0.259^*^	0.735^**^	0.747^**^	0.678^**^	1					
6. lo_conc_image	0.180	0.769^**^	0.725^**^	0.719^**^	0.149	1				
7. hi_image_image	0.199	0.567^**^	0.609^**^	0.463^**^	0.954^**^	−0.040	1			
8. lo_image_image	0.188	0.770^**^	0.708^**^	0.761^**^	0.155	0.982^**^	−0.056	1		
9. hi_AoA_image	0.254^*^	0.835^**^	0.742^**^	0.941^**^	0.506^**^	0.720^**^	0.264^*^	0.794^**^	1	
10. lo_AoA_image	0.230	0.826^**^	0.863^**^	0.698^**^	0.947^**^	0.338^**^	0.907^**^	0.312^**^	0.510^**^	1

* *Correlation is significant at the 0.05 level (2-tailed)*.

** *Correlation is significant at the 0.01 level (2-tailed)*.

### Relationship Between L2 Proficiency, Frequency, Concreteness, and Imageability

**Figure 1:**
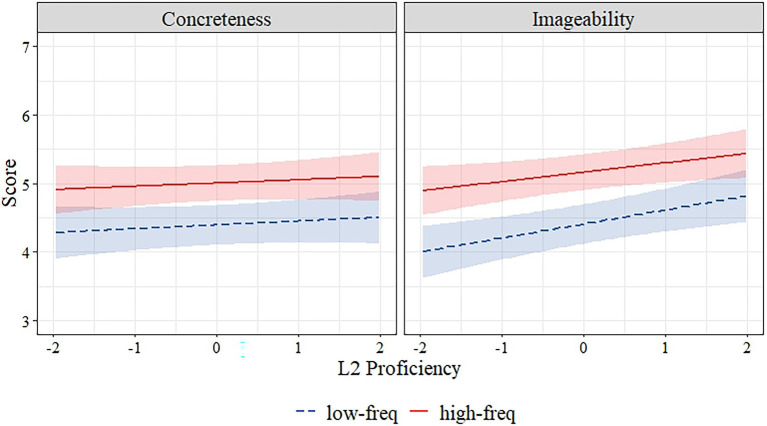
The relationship between L2 proficiency and scaled scores of concreteness, imageability ratings of hi-lo frequency words. The hues around regression lines represent 95% confidence intervals.

In order to investigate the relation between L2 proficiency, frequency, concreteness, and imageability, we fitted a linear mixed-effects model in R (R Core Team, 2021) using the lmer() function from the lme4 library (Bates et al., 2015). In the model rating type (concreteness–imageability), frequency (high–low) and L2 proficiency were the fixed factors, and lexical ratings (for concreteness and imageability) were the outcome variable. We took subject and word as random intercepts in order to control for subject variability and word variability (which incorporates a specific word being high or low on concreteness and imageability). We centered all the fixed factors to avoid convergence problems in the model. We used the lmerTest package (Kuznetsova et al., 2017) in R to obtain *p* values for the fixed effects. All model estimates are presented in Table 5. The model revealed significant fixed effect of rating type, frequency, as well as a significant interaction of rating type × frequency × L2 proficiency. L2 proficiency was not a significant fixed effect. However, the significant three-way interaction indicates that the association between frequency and imageability is unique. We used *sim_slopes* function to probe simple slope estimates for interactions with continuous predictor (L2-proficiency). Again results indicated that L2 proficiency was associated with increased imageability ratings by 0.20 ± 0.06 a only for low-frequency words *t* = 3.26, *p* < 0.001. Significant interaction is presented in Figure 1.

**Table 5:** Linear-mixed effects regression model summary for frequency, imageability, and concreteness.

	Coefficient	SE	*t* value	*p*	
**Fixed effects**					
Intercept	4.393	0.063	29.84	<0.001	^***^
L2 proficiency	0.056	0.006	0.89	0.38	
Frequency (hi/lo)	0.612	0.036	3.44	<0.001	^***^
Rating type (concreteness/imageability)	0.015	0.021	0.72	0.46	
L2 proficiency * frequency * rating type	−0.059	0.002	−2.17	0.03	^*^
**Random effects**	Variance	SD			
Subject Intercept	0.27	0.52			
Word Intercept	2.71	1.65			

*** *p < 0.001*;

* *p < 0.05*.

### Relationship Between L2 Proficiency, AoA, Concreteness, and Imageability

**Figure 2:**
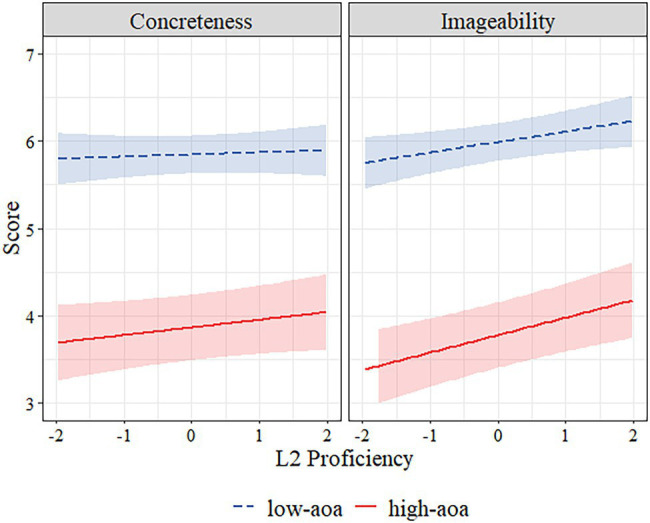
The relationship between L2 proficiency and scaled scores of concreteness, imageability ratings of hi-lo AoA words. The hues around regression lines represent 95% confidence intervals.

In order to investigate the relation between L2 Proficiency, AoA, concreteness, and imageability, we again fitted a linear mixed-effects model in R (R Core Team, 2021) using the lmer() function from the lme4 library (Bates et al., 2015). In this model, rating type (concreteness–imageability), AoA (high–low), and L2 proficiency were the fixed factors, and lexical ratings (for concreteness and imageability) were the outcome variable. We again entered subject and word as random intercepts. We again centered all the fixed factors. We used the lmerTest package (Kuznetsova et al., 2017) in R to obtain p values for the fixed effects. All model estimates are presented in Table 6. The model revealed a significant fixed effect of rating type, AoA, and a significant interaction of rating type × AoA × L2 proficiency. L2 proficiency was again not a significant predictor as a fixed effect. There was a significant three-way interaction of AoA, L2 proficiency, and rating type, which indicates the association between AoA and imageability. We ran simple slope analyses to reveal estimates for the interaction effect. Again results indicated that L2 proficiency was positively associated with imageability ratings only for high-AoA words (*β* = 0.12, SE = 0.05, *p* = 0.02; See Figure 2; Table 6).

**Table 6:** Linear-mixed effects regression model summary for AoA, imageability, and concreteness.

	Coefficient	SE	*t* value	*p*	
**Fixed effects**					
Intercept	5.847	0.110	53.03	<0.001	^***^
L2 proficiency	0.024	0.051	0.47	0.63	
AoA (hi/lo)	−1.982	0.209	−9.46	<0.001	^***^
Rating type (concreteness/imageability)	0.143	0.018	7.96	<0.001	^***^
L2 proficiency * AoA * rating type	0.063	0.027	2.33	0.02	^*^
**Random effects**	Variance	SD			
Subject Intercept	0.17	0.42			
Word Intercept	1.61	1.27			

*** *p < 0.001*;

* *p < 0.05*.

## Discussion

The first goal of this study was to reveal the significance of bilingualism reflected in L2 proficiency on lexical property evaluations. Previous research found that these lexical properties influence several task performances and that they are closely related to each other. However, perceiving and representing the words in an additional language and its consequences lexical evaluations did not receive much attention in the literature. In order to examine the relationship between lexical property evaluations, frequency, AoA, and L2 proficiency, we computed eight different subscores for each concreteness and imageability ratings based on high/low frequency, high/low concreteness, high/low imageability, and high/low AoA.

We asked whether (1) lexical properties such as frequency, concreteness, and imageability, AoA are related to each other, and (2) whether L2 proficiency is associated with this relationship. We computed correlations as preliminary analysis based on different subscores; we then computed two models using linear mixed-effects regression analyses. The first model investigated the unique relations between the L2 proficiency, frequency, and lexical ratings. The model incorporated subjective ratings (for concreteness and imageability) of participants as outcome variables while taking into account fixed effects of L2 proficiency, rating type (either concreteness or imageability), and interaction effect of L2 proficiency × frequency × rating type. We also added subject and word as random intercepts. The significant interaction effect followed by simple slope analyses revealed a positive relationship between L2 proficiency and imageability ratings only for low-frequency words. The second model tested by linear mixed-effects regression analyses investigated the relations between the L2 proficiency, AoA, and lexical ratings. This model again incorporated subjective ratings (for concreteness and imageability) of participants as our dependent variable, with fixed effects of L2 proficiency, rating type (either concreteness or imageability), and interaction effect of L2 proficiency × AoA × rating type. We again added subject and word as a random intercept. Results indicated that L2 proficiency was positively related with imageability ratings but for only low-frequency words and later acquired words and not for others. Thus, the unique relationships of L2 proficiency were confirmed for both frequency and AoA. It is worth noting that the results indicated a significant but weak relationship due to the small effect size. However, this weak relationship might be due to the fact that imageability ratings might be related to additional factors other than L2 proficiency.

Overall, our findings for imageability were in line with the interaction of bilingual representation under the Dual-coding theory. Dual-coding theory suggests that bilinguals have two verbal representations connected to the imaginal representation, which may lead to better imageability for bilinguals than monolinguals (Paivio and Desrochers, 1980). Hence, as L2 proficiency increased, the imageability also increased. These findings might be considered within the context of the Shared Asymmetrical Model (Dong, et al., 2005). This model proposes that some concepts might be more salient in some languages and that L1 and L2 share common and independent stores. Thus, adding an L2 in someone’s repertoire might enhance the salience of some lexical concepts and thus reflect on lexical evaluations. Our results might also be related to Pavlenko’s (2009) the Modified Hierarchical Model. The model proposes the developmental dimension is influential in forming lexical links between L1 and L2. As L2 learners develop through lexical to conceptual mediation, L2 proficiency, as well as AoA, suggest key roles. Our findings also contribute to this literature in highlighting the importance of these concepts. Specifically, our results for relations between frequency/L2 proficiency and AoA/L2 proficiency and imageability points to the fact that frequency and AoA dimensions should be taken into account separately. The present study involved AoA and frequency of L1 words; however, our results also point to future directions about these concepts taken into account for L2 as well. In other words, the acquisition of words might be following different orders for L2 as high-frequency words might not be always acquired earlier. Therefore, the interplay between L1 and L2 is more complex than that might have been considered.

Although there is a relation between the L2 proficiency and imageability, the strength of the association is not high. This might be due to possible differentiation of imagery ability and other individual differences between participants in addition to their second language proficiency variance. Imagery is a multidimensional experience comprising several aspects. Evaluating the words in terms of imageability not only requires the ability to process words and evaluate them within a scale but also requires a visual retrieval that is related to their visual and memory capacities. While re-generating and imaging the words, individuals can use various operations reflecting their verbal and visual capabilities. For instance, greater imagery strength was positively associated with greater visual working memory (Keogh and Pearson, 2014). The relationship between reported imagery vividness and individual differences is found to be related to many aspects, including background experience in a related activity such as sports (Di Corrado et al., 2014), music (Aleman et al., 2000), and video games (Floridou et al., 2021). Especially for less frequent words, the individual differences between vocabulary and world knowledge word processing speed might be another underlying factor for the imageability ratings. Briefly, since the only factor is not L2 proficiency during the evaluation of lexical properties, the relationship between L2 proficiency and imageability might be lower than expected. Future studies should investigate other possible explanations that could relate to individual differences demographic factors between the participants.

Contrary to the significant association between L2 proficiency and imageability, concreteness ratings were not significantly correlated with L2 proficiency. One possible explanation of the lack of association between L2 proficiency and concreteness might be since concreteness might be more inherent and might not be open to subjective evaluation as much as imageability. Thus, in early works, concreteness was regarded as a feature of the lexical organization but not an indication of imageability of the verbal material (Richardson, 1975). The results of the linear mixed-effects analysis enabled us to confirm the relation between imageability and L2 is indeed unique and evident.

Although our results show that imageability and concreteness are positively associated in line with the previous findings (Paivio et al., 1968; Yao et al., 2018), L2 proficiency was not found to be positively related to concreteness while it was for imageability. This result addresses previous literature on the similarity and distinction between concreteness and imageability. As stated, while some authors used these two concepts interchangeably, Paivio et al. (1968) stated that they are, in fact dissociable despite the positive correlation between the ratings. This dissociation is based on the argument that the nature of two concepts differs from each, so that they cannot be used alternatively since concreteness is more categorical when it is compared to imageability, which can be rated along a scale. This implies that there may be words that are not concrete but imageable. Although concrete items may easily evoke an image in mind than abstract items (Altarriba and Bauer, 2004), despite being regarded as abstract, some words are found to be highly imageable such as “affection” and “anger” which arouse emotions (Paivio et al., 1968). In our data, we found a similar trend where emotionally laden words tend to have higher imageability ratings than their concreteness ratings (e.g., *mutluluk* “happiness,” *korku* “fear”) which can also be confirmed by the Turkish Word Norms (Tekcan and Göz, 2005).

Another underlying reason might be the argument that concreteness is “just a feature of lexical organization” while imageability reflects the image-evoking property of words (Richardson, 1975). One other explanation for lack of association between concreteness as L2 proficiency might be related to the fact that representations of concrete words are shared largely by languages; therefore, this might not be prone to L2 proficiency. Additionally, in our stimuli of 600 words, there were 337 high concrete words and 147 low concrete words, which implies the dominance of high concrete words over low concrete words on overall basis.

We found a positive relationship between L2 proficiency and imageability ratings for low-frequency words but not for high-frequency words. The reason why participants had higher imageability as L2 proficiency increased for low-frequency words but not for high-frequency words might be explained by the weaker link hypothesis (Gollan et al., 2008), which proposes that bilingual disadvantage stem from dividing frequency-of-use between two languages. According to this hypothesis, low-frequency words are more prone to be affected by L2 proficiency (Gollan et al., 2008). Since dividing frequency weakens the link with the words for bilinguals, low-frequency words might be affected by this division more than high-frequency words, which have stronger links that are harder to change.

There was a positive relationship between L2 proficiency and imageability ratings for high-AoA words but not for low-AoA words. The lack of association between L2 proficiency and imageability for low-AoA words might be explained by learning an L2 after age five for all participants. Thus, the effect of the exposure to words in L2 was more powerful for the later acquired words. This finding might also be related to the nature of the interaction between frequency and AoA. As many researchers suggest, early acquired words are also commonly used later in life, leading to a higher frequency (Gerhand and Barry, 1998). Thus, in line with this view, our two findings demonstrated a positive relationship between L2 proficiency and imageability for both low-frequency words and concordantly for later acquired words. Additionally, data from several studies show that AoA can be associated with low-frequency words but not with high-frequency words (Bonin et al., 2002; Brysbaert et al., 2016), which can also be another factor that might have impacted our results. The common ground for these two variables might be having weaker mapping than high-frequency words and early acquired words, which makes any change in representation easier. These findings might suggest that L2 proficiency would be linked to imageability, and this link is also connected with other psycholinguistic variables that may catalyze or suppress the connection. While imageability is positively related with L2 proficiency, this relationship is not independent of the frequency ratings and AoA of target words as previously suggested by some researchers (Bonin et al., 2004; Brysbaert et al., 2016; Göz et al., 2017).

Our results also reflect on the literature that focuses on the relationship between lexical properties and task performance. Many studies examine how lexical properties such as frequency, word length, concreteness, and imageability may affect task performance and be related to one another. This study not only confirms the previous findings demonstrating the influence of AoA and frequency on word evaluations (Gerhand and Barry, 1998; Bonin et al., 2002) but also shows that L2 proficiency is another crucial variable that should be taken into account. Thereafter, L2 should be controlled for future studies that require lexical processing.

Working with verbal stimuli has many factors that can make a difference in the task performance which makes psycholinguistic variables (e.g., frequency, word length, concreteness, and imageability) remarkable in psychological research. Although previous studies revealed the importance of frequency, AoA, and concreteness in processing words faster or more accurately (Carroll and White, 1973; Forster and Chambers, 1973; Whaley, 1978), the current study extends this information with their relationship between L2 proficiency and how learning an L2 is related with L1 lexical property evaluation. Based on our findings, individuals with higher L2 proficiency have more enriched view reflected in higher imageability ratings over less proficient ones. Although we directly did not test any bilingual lexicon model, the Modified Hierarchical Model (Pavlenko, 2009) suggests as L2 proficiency increases, there will be greater overlap between conceptual representation, which further implies an enriched conceptualization. Looking from a broader perspective, studies showing the association between improved comprehension by L2 acquisition support the idea that learning a new language may provide us with a more enriched worldview.

In addition to positive findings, lack of relationship would also provide insight for future research. Not finding an association between L2 proficiency and concreteness unlike the imageability, might tell us that we should consider and control the two properties separately. Using the two terms interchangeably and assuming the same results might be misleading. Although they are correlated, imageability does not always predict concreteness as we mentioned above (i.e., emotion-laden words).

We did not find any main effect of L2 proficiency in the models we have tested, however, we found the interaction effects which points to where L2 proficiency is influential. Since our sample consisted of adults having diverse range of proficiency (e.g., PPVT-IV scores between 43 to 199), we think this result is not a consequence of having a non-diverse group. We think L2 proficiency affects to certain extents and for certain contexts. In order to reveal the effects of L2 proficiency on lexical property evaluations in greater detail, future research should focus on experimental designs, especially by comparing monolingual and bilingual groups. Although we did not compare these two groups, within bilinguals which we recruited, there was a diverse range of L2 proficiency.

In conclusion, the unique relation between L2 proficiency and imageability is strengthened by running several analyses to test our predictions. This study contributes to the literature by emphasizing the importance of L2 proficiency for research where lexical properties are taken into account.

## Data Availability Statement

The raw data supporting the conclusions of this article will be made available by the authors, without undue reservation.

## Ethics Statement

The studies involving human participants were reviewed and approved by Kadir Has University - Human Studies Ethical Board. The patients/participants provided their written informed consent to participate in this study.

## Author Contributions

All authors listed have made a substantial, direct, and intellectual contribution to the work and approved it for publication.

## Conflict of Interest

The authors declare that the research was conducted in the absence of any commercial or financial relationships that could be construed as a potential conflict of interest.

## Publisher’s Note

All claims expressed in this article are solely those of the authors and do not necessarily represent those of their affiliated organizations, or those of the publisher, the editors and the reviewers. Any product that may be evaluated in this article, or claim that may be made by its manufacturer, is not guaranteed or endorsed by the publisher.

## References

[ref1] Aleman A. Nieuwenstein M. R. Böcker K. B. de Haan E. H. F. (2000). Music training and mental imagery ability. Neuropsychologia 38, 1664–1668. doi: 10.1016/s0028-3932(00)00079-8, PMID: 11074089

[ref2] Alonso M. A. Fernandez A. Díez E. (2011). Oral frequency norms for 67,979 Spanish words. Behav. Res. Methods 43, 449–458. doi: 10.3758/s13428-011-0062-3, PMID: 21416306

[ref100] Altarriba J. Bauer L. M. (2004). The distinctiveness of emotion concepts: A comparison between emotion, abstract, and concrete words. Am. J. Psychol. 117, 389–410. doi: 10.2307/4149007 15457808

[ref3] Altarriba J. Bauer L. M. Benvenuto C. (1999). Concreteness, context availability, and imageability ratings and word associations for abstract, concrete, and emotion words. Behav. Res. Methods Instrum. Comput. 31, 578–602. doi: 10.3758/BF03200738, PMID: 10633977

[ref4] Arnon I. McCauley S. M. Christiansen M. H. (2017). Digging up the building blocks of language: age-of-acquisition effects for multiword phrases. J. Mem. Lang. 92, 265–280. doi: 10.1016/j.jml.2016.07.004

[ref5] Baddeley A. D. Thomson N. Buchanan M. (1975). Word length and the structure of short-term memory. J. Verbal Learn. Verbal Behav. 14, 575–589. doi: 10.1016/S0022-5371(75)80045-4

[ref6] Balota D. A. Chumbley J. I. (1984). Are lexical decisions a good measure of lexical access? The role of word frequency in the neglected decision stage. J. Exp. Psychol. Hum. Percept. Perform. 10, 340–357. doi: 10.1037/0096-1523.10.3.340, PMID: 6242411

[ref7] Barry C. Morrison C. M. Ellis A. W. (1997). Naming the Snodgrass and Vanderwart pictures: effects of age of acquisition, frequency and name agreement. Q. J. Exp. Psychol. 50, 560–585. doi: 10.1080/783663595

[ref8] Bates D. Maechler M. Bolker B. Walker S. (2015). Fitting linear mixed-effects models using lme4. J. Stat. Softw. 67, 340–357. doi: 10.18637/jss.v067.i01

[ref9] Bird H. Franklin S. Howard D. (2001). Age of acquisition and imageability ratings for a large set of words, including verbs and function words. Behav. Res. Methods Instrum. Comput. 33, 73–79. doi: 10.3758/BF03195349, PMID: 11296722

[ref10] Bonin P. Barry C. Méot A. Chalard M. (2004). The influence of age of acquisition in word reading and other tasks: a never ending story? J. Mem. Lang. 50, 456–476. doi: 10.1016/j.jml.2004.02.001

[ref11] Bonin P. Chalard M. Méot A. Fayol M. (2002). The determinants of spoken and written picture naming latencies. Br. J. Psychol. 93, 89–114. doi: 10.1348/000712602162463, PMID: 11839103

[ref12] Brysbaert M. Mandera P. Keuleers E. (2018). The word frequency effect in word processing: an updated review. Curr. Dir. Psychol. Sci. 27, 45–50. doi: 10.1177/0963721417727521

[ref13] Brysbaert M. Stevens M. Mandera P. Keuleers E. (2016). The impact of word prevalence on lexical decision times: evidence from the Dutch lexicon project 2. J. Exp. Psychol. Hum. Percept. Perform. 42, 441–458. doi: 10.1037/xhp0000159, PMID: 26501839

[ref14] Brysbaert M. Warriner A. B. Kuperman V. (2014). The word frequency effect in word processing: an updated review. Behav. Res. Methods 46, 904–911. doi: 10.3758/s13428-013-0403-5 24142837

[ref15] Butler B. Hains S. (1979). Individual differences in word recognition latency. Mem. Cogn. 7, 68–76. doi: 10.3758/BF03197587

[ref16] Carroll J. B. White M. N. (1973). Word frequency and age of acquisition as determiners of picture-naming latency. Q. J. Exp. Psychol. 25, 85–95. doi: 10.1080/14640747308400325

[ref18] Connell L. Lynott D. (2012). Strength of perceptual experience predicts word processing performance better than concreteness or imageability. Cognition 125, 452–465. doi: 10.1016/j.cognition.2012.07.010 22935248

[ref20] Cortese M. J. McCarty D. P. Schock J. (2015). A mega recognition memory study of 2897 disyllabic words. Q. J. Exp. Psychol. 68, 1489–1501. doi: 10.1080/17470218.2014.945096, PMID: 25220011

[ref21] Cortese M. J. Schock J. (2013). Imageability and age of acquisition effects in disyllabic word recognition. Q. J. Exp. Psychol. 66, 946–972. doi: 10.1080/17470218.2012.722660, PMID: 23030642

[ref22] Davies S. K. Izura C. Socas R. Dominguez A. (2016). Age of acquisition and imageability norms for base and morphologically complex words in English and in Spanish. Behav. Res. Methods 48, 349–365. doi: 10.3758/s13428-015-0579-y, PMID: 25939978

[ref23] De Groot A. M. Dannenburg L. Vanhell J. G. (1994). Forward and backward word translation by bilinguals. J. Mem. Lang. 33, 600–629.

[ref24] Desrochers A. Bergeron M. (2000). Valeurs de fréquence subjective et d’imagerie pour unéchantillon de 1,916 substantifs de la langue française. Can. J. Exp. Psychol. 54, 274–325. doi: 10.1037/h0087347 11195718

[ref25] Desrochers A. Thompson G. L. (2009). Subjective frequency and imageability ratings for 3,600 French nouns. Behav. Res. Methods 41, 546–557. doi: 10.3758/BRM.41.2.546, PMID: 19363197

[ref26] Di Corrado D. Guarnera M. Quartiroli A. (2014). Vividness and transformation of mental images in karate and ballet. Percept. Mot. Skills 119, 764–773. doi: 10.2466/22.24.PMS.119c30z6, PMID: 25456250

[ref200] Dong Y. Gui S. MacWhinney B. (2005). Shared and separate meanings in the bilingual mental lexicon. Bilingualism: Language and Cognition 8, 221–238. doi: 10.1017/S1366728905002270, PMID: 27743317

[ref27] Duyck W. Vanderelst D. Desmet T. Hartsuiker R. J. (2008). The frequency effect in second-language visual word recognition. Psychon. Bull. Rev. 15, 850–855. doi: 10.3758/PBR.15.4.850, PMID: 18792515

[ref28] Elsherif M. M. Catling J. C. Frisson S. (2019). Two words as one: a multi-naming investigation of the age-of-acquisition effect in compound-word processing. Mem. Cogn. 48, 511–525. doi: 10.3758/s13421-019-00986-6, PMID: 31755026 PMC7242258

[ref29] Fliessbach K. Weis S. Klaver P. Elger C. E. Weber B. (2006). The effect of word concreteness on recognition memory. NeuroImage 32, 1413–1421. doi: 10.1016/j.neuroimage.2006.06.007 16861011

[ref30] Floridou G. A. Peerdeman K. J. Schaefer R. S. (2021). Individual differences in mental imagery in different modalities and levels of intentionality. Mem. Cogn. 50, 1–16. doi: 10.3758/s13421-021-01209-7 PMC8763825 34462893

[ref31] Forster K. I. Chambers S. M. (1973). Lexical access and naming time. J. Verbal Learn. Verbal Behav. 12, 627–635. doi: 10.1016/S0022-5371(73)80042-8

[ref32] Gerhand S. Barry C. (1998). Word frequency effects in oral reading are not merely age-of- acquisition effects in disguise. J. Exp. Psychol. Learn. Mem. Cogn. 24, 267–283. doi: 10.1037/0278-7393.24.2.267

[ref33] Gernsbacher M. A. (1984). Resolving 20 years of inconsistent interactions between lexical familiarity and orthography, concreteness, and pol-ysemy. J. Exp. Psychol. Gen. 113, 256–281. doi: 10.1037/0096-3445.113.2.256, PMID: 6242753 PMC4311894

[ref34] Gilhooly K. J. Logie R. H. (1980). Age-of-acquisition, imagery, concreteness, familiarity, and ambiguity measures for 1,944 words. Behav. Res. Methods Instrum. 12, 395–427. doi: 10.3758/BF03201693

[ref35] Gilhooly K. J. Logie R. H. (1981). Word age-of-acquisition and visual recognition thresholds. Curr. Psychol. Res. 1, 215–225. doi: 10.1007/BF03186732

[ref36] Gollan T. H. Montoya R. I. Cera C. Sandoval T. C. (2008). More use almost always means a smaller frequency effect: aging, bilingualism, and the weaker links hypothesis. J. Mem. Lang. 58, 787–814. doi: 10.1016/j.jml.2007.07.001, PMID: 19343088 PMC2409197

[ref37] Göz I. (2003). Yazılı Türkçenin Kelime Sıklığı Sözlüğü [Word Frequency Dictionary of Written Turkish] Ankara, Turkey: Türk Dil Kurumu

[ref38] Göz İ. Tekcan A. İ. Erciyes A. A. (2017). Subjective age-of-acquisition norms for 600 Turkish words from four age groups. Behav. Res. Methods 49, 1736–1746. doi: 10.3758/s13428-016-0817-y, PMID: 27743317

[ref39] Grosjean F. Li P. (2013). The Psycholinguistics of Bilingualism. New York, NY: Wiley-Blackwell.

[ref40] Holmes V. M. Langford J. (1976). Comprehension and recall of abstract and concrete sentences. J. Verbal Learn. Verbal Behav. 15, 559–566. doi: 10.1016/0022-5371(76)90050-5

[ref41] Ivanova I. Costa A. (2008). Does bilingualism hamper lexical access in speech production? Acta Psychol. 127, 277–288. doi: 10.1016/j.actpsy.2007.06.003, PMID: 17662226

[ref42] Izura C. Pérez M. A. Agallou E. Wright V. C. Marín J. Stadthagen-González H.. (2011). Age/order of acquisition effects and the cumulative learning of foreign words: a word training study. J. Mem. Lang. 64, 32–58. doi: 10.1016/j.jml.2010.09.002

[ref43] James C. T. (1975). The role of semantic information in lexical decisions. J. Exp. Psychol. Hum. Percept. Perform. 104, 130–136.

[ref44] Johnson C. J. Paivio A. Clark J. M. (1996). Cognitive components of picture naming. Psychol. Bull. 120, 113–139. doi: 10.1037/0033-2909.120.1.113 8711012

[ref45] Kecskes I. Papp T. (2003). “How to demonstrate the conceptual effect of the L2 on L1?” in The Effect of L2 on L1. ed. Cook V. (Clevedon: Multilingual Matters), 247–267.

[ref46] Keogh R. Pearson J. (2014). The sensory strength of voluntary visual imagery predicts visual working memory capacity. J. Vis. 14, 7–7. doi: 10.1167/14.12.7, PMID: 25301015

[ref300] Kroll J. F. Stewart E. (1994). Category interference in translation and picture naming: Evidence for asymmetric connections between bilingual memory representations. J. Exp. Child. Psychol. 33, 149–174. doi: 10.1006/jmla.1994.1008

[ref47] Kuznetsova A. Brockhoff P. B. Christensen R. H. B. (2017). lmerTest package: tests in linear mixed effects models. J. Stat. Softw. 82, 1–26. doi: 10.18637/jss.v082.i13

[ref48] McMullen P. A. Bryden M. P. (1987). The effects of word imageability and frequency on hemispheric asymmetry in lexical decisions. Brain Lang. 31, 11–25. doi: 10.1016/0093-934X(87)90057-5

[ref49] Meyer D. E. Schvaneveldt R. W. (1971). Facilitation in recognizing pairs of words: evidence of a dependence between retrieval operations. J. Exp. Psychol. 90, 227–234. doi: 10.1037/h0031564, PMID: 5134329

[ref50] Morrison C. M. Conway M. A. (2010). First words and first memories. Cognition 116, 23–32. doi: 10.1016/j.cognition.2010.03.011 20363469

[ref51] Morrison C. M. Ellis A. W. (1995). Roles of word frequency and age of acquisition in word naming and lexical decision. J. Exp. Psychol. Learn. Mem. Cogn. 21, 116–133. doi: 10.1037/0278-7393.21.1.116

[ref52] Paivio A. (1971). Imagery and Verbal Processes. Holt, Rinehart & Winston: Psychology Press.

[ref53] Paivio A. Desrochers A. (1980). A dual-coding approach to bilingual memory. Canadian J. Psychol. 34, 388–399. doi: 10.1037/h0081101

[ref54] Paivio A. Yuille J. C. Madigan S. A. (1968). Concreteness, imagery, and meaningfulness values for 925 nouns. J. Exp. Psychol. 76, 1–25. doi: 10.1037/h0025327, PMID: 5672258

[ref55] Pavlenko A. (2003). “Chapter 3. ‘I feel clumsy speaking Russian’: L2 influence on L1 in narratives of Russian L2 users of English,” in Effects of the Second Language on the First. ed. Cook V. (Bristol, Blue Ridge Summit: Multilingual Matters), 32–61.

[ref56] Pavlenko A. (2009). “Conceptual representation in the bilingual lexicon and second language vocabulary learning,” in The Bilingual Mental Lexicon: Interdisciplinary Approaches. ed. Pavlenko A. (Clevedon: Multilingual Matters), 125–160.

[ref500] Potter M. C. So K. F. Von Eckardt B. Feldman L. B. (1984). Lexical and conceptual representation in beginning and proficient bilinguals. Journal of verbal learning and verbal behavior 23, 23–38. doi: 10.1016/S0022-5371(84)90489-4, PMID: 27743317

[ref600] R Core Team (2021). R: A language and environment for statistical computing. R Foundation for Statistical Computing. Vienna, Austria. Available at: https://www.R-project.org/

[ref57] Ransdell S. E. Fischler I. (1989). Effects of concreteness and task context on recall of prose among bilingual and monolingual speakers. J. Mem. Lang. 28, 278–291. doi: 10.1016/0749-596X(89)90034-X

[ref58] Reilly J. Kean J. (2007). Formal distinctiveness of high and low imageability nouns: analyses and theoretical implications. Cogn. Sci. 31, 157–168. doi: 10.1080/03640210709336988, PMID: 21635291

[ref59] Richardson J. T. E. (1975). Concreteness and Imageability. Q. J. Exp. Psychol. 27, 235–249. doi: 10.1080/14640747508400483 1196589

[ref60] Schwanenflugel P. J. Harnishfeger K. K. Stowe R. W. (1988). Context availability and lexical decisions for abstract and concrete words. J. Mem. Lang. 27, 499–520. doi: 10.1016/0749-596X(88)90022-8

[ref61] Shimron J. Chernitsky R. (1995). Typicality shift in semantic categories as a result of cultural transition: evidence from Jewish argentine immigrants in Israel. Discourse Process. 19, 265–278. doi: 10.1080/01638539509544917

[ref63] Tekcan A. İ. Göz İ. (2005). Türkçe kelime normları [Turkish Word norms]. İstanbul, Turkey: Boğaziçi Üniversitesi Yayınevi.

[ref400] Van Hell J. G. De Groot A. M. (1998). Conceptual representation in bilingual memory: Effects of concreteness and cognate status in word association. Bilingualism: Language and cognition. 1, 193–211. doi: 10.1017/S1366728998000352

[ref64] van Hell J. G. Dijkstra T. (2002). Foreign language knowledge can influence native language performance in exclusively native contexts. Psychon. Bull. Rev. 9, 780–789. doi: 10.3758/BF03196335, PMID: 12613683

[ref65] Walker I. Hulme C. (1999). Concrete words are easier to recall than abstract words: evidence for a semantic contribution to short-term serial recall. J. Exp. Psychol. Learn. Mem. Cogn. 25, 1256–1271.

[ref66] Whaley C. P. (1978). Word—nonword classification time. J. Verbal Learn. Verbal Behav. 17, 143–154. doi: 10.1016/S0022-5371(78)90110-X

[ref67] Yao Z. Wu J. Zhang Y. Wang Z. (2018). Norms of valence, arousal, concreteness, familiarity, imageability, and context availability for 1, 100 Chinese words. Behav. Res. Methods 49, 1374–1385. doi: 10.3758/s13428-016-0793-2, PMID: 27553483

