# Supplementary material for: Relations Between L2 Proficiency and L1 Lexical Property Evaluations
Source: Front Psychol. 2022 Mar 17;13:820702. doi: 10.3389/fpsyg.2022.820702 (PMC8968421; doi:10.3389/fpsyg.2022.820702)
Supplement: Supplementary file 1 [file Table_1.DOCX]

Supplementary Material

# Imageability and Concreteness Ratings

Participants were instructed to complete two evaluations as follows:

*Instruction for Concreteness Rating:*

In this study, we expect you to make a decision that you find appropriate for the words you see in the presented list. Your decision for a word could be ***abstract***, ***concrete,*** or between the two. A concrete word is a word that can be a real object, a material, or a person that can be perceived by sense organs. An abstract word, on the other hand, is a word that denotes a real or imaginary entity or relationship that cannot be perceived with the sense organs. For this purpose, you will use a 7-point scale.

1----------------2----------------3----------------4----------------5----------------6----------------7

This is a concreteness scale. Once you have decided how ***concrete*** or ***abstract*** the words are, you need to click on the appropriate number for your decision. For example, you can use the number 6 or 7 (Concrete) for the word "Chair" and the number 1 or 2 (Abstract) for the word "Betrayal". For the words you think do not clearly belong to either ***concrete*** or ***abstract*** categories, you can evaluate the words with a rating you find appropriate on the scale between ***concrete*** and ***abstract*** ends, according to your own assessment.

*Instruction for Imageability Rating:*

Words differ in their capacity to evoke images (imageability). Some words quickly evoke sensory experiences, such as mental images or sounds. Whereas, others can only evoke mental images after a while (i.e., harder) or not at all. The purpose of this study is to rate the mental imageability capacities of the words in the presented list. If a word, in your opinion, quickly and easily evokes a mental image (i.e., this image can be visual, auditory, or any other form), you should give that word a ***high imageability*** rating. If the word barely evokes an image or does not evoke at all, you should give that word a ***low imageability*** rating. For example, consider the words “Apple” or “Time”. “Apple” evokes images quite quickly and easily. Therefore, a ***high imageability*** rating should be given. But it is very difficult for the word 'Time' to evoke an image. Therefore, a ***low imageability*** score should be given. There is always a possibility that any word on the list reminds another word that is closely related to that word but is not on the list. For instance, the word “Fork” can evoke the word “Knife” that is not on the list. In such circumstances, rate the word on the list, in this case “Fork” but not the word that it evokes, which is “Knife”.

You will make your rating on a 7-point scale. The end of this scale marked with 1 indicates low image status, and the end marked with 7 indicates high image status. Choose the rating that you think best describes your decision for the imageability rating of the word you are evaluating. For instance, you should choose 7 for the words like “Apple”, that you think to evoke the quickest and easiest imagery. For the words that you have difficulty imagining or cannot imagine at all, for example like “Time”, you should choose 1 as the appropriate rating. If the degree of imageability is between the two extremes, depending on the ease or difficulty of imagining, choose between 2 to 6, depending on how appropriate you think the rating is. The rating you will choose from 1 to 7 depends solely on your own judgment and decision. Please be very careful with your decisions and definitely do not rush. In order to give you an idea, the rating of five words is given below:

1----------------2----------------3----------------4----------------5----------------6----------------7

democracy                                     funny           writer           steam        automobile
